# Communicative Interactions Improve Visual Detection of Biological Motion

**DOI:** 10.1371/journal.pone.0014594

**Published:** 2011-01-26

**Authors:** Valeria Manera, Cristina Becchio, Ben Schouten, Bruno G. Bara, Karl Verfaillie

**Affiliations:** 1 Department of Psychology, Center for Cognitive Science, University of Turin, Turin, Italy; 2 Laboratory of Experimental Psychology, Department of Psychology, Katholieke Universiteit Leuven, Leuven, Belgium; University of Minnesota, United States of America

## Abstract

**Background:**

In the context of interacting activities requiring close-body contact such as fighting or dancing, the actions of one agent can be used to predict the actions of the second agent [1]. In the present study, we investigated whether interpersonal predictive coding extends to interactive activities – such as communicative interactions - in which no physical contingency is implied between the movements of the interacting individuals.

**Methodology/Principal Findings:**

Participants observed point-light displays of two agents (A and B) performing separate actions. In the communicative condition, the action performed by agent B responded to a communicative gesture performed by agent A. In the individual condition, agent A's communicative action was substituted with a non-communicative action. Using a simultaneous masking detection task, we demonstrate that observing the communicative gesture performed by agent A enhanced visual discrimination of agent B.

**Conclusions/Significance:**

Our finding complements and extends previous evidence for interpersonal predictive coding, suggesting that the communicative gestures of one agent can serve as a predictor for the expected actions of the respondent, even if no physical contact between agents is implied.

## Introduction

Dancing a waltz, playing a piano duet, carrying a table together are all examples of joint activities requiring a considerable degree of interpersonal coordination. To successfully engage in these activities, actors must be able to direct their attention to where the interaction partner is attending (joint attention) [2], [3] and to adjust their actions to those other persons choosing an appropriate complementary action to be performed at an appropriate time [4]. Time places serious constraints on joint actions and, as the time windows for coordination are often very narrow, actors must achieve a close temporal coordination for acting synchronously or in turns [5]. Similarly, to avoid bumping into one another or into an obstacle (e.g. when carrying an object together), they need to effectively distribute a common space and optimize movement paths. Under these circumstances, the possibilities for moving and for completing actions are jointly constrained [6]. As dynamical principles constrain the coordination of interpersonal movements, the actions of one agent can serve as predictors for the expected actions of the other agent [7]. Neri, Luu, and Levy [1] have indeed demonstrated that in the context of interacting activities requiring close-body contact such as fighting or dancing, the actions of one agent can be used to guide the processing of the actions performed by the other agent. Participants observed point-light displays of two fighters masked with noise dots scattered all over the screen. Visual detection of the target agent was better when the agent was embedded in a fighting sequence with the second agent acting synchronously as opposed to asynchronously, even though synchronization was irrelevant to the visual discrimination task. These findings suggest that implicit knowledge about the natural dynamics of human interaction guides the processing of motion patterns generated by the actions of individual agents. Because the actions of the two agents are dynamically coupled, the action of one agent can be used to predict the action of the other agent. In the present study, we investigated whether interpersonal predictive coding extends to interactive activities in which no physical contingency is implied between the movements of the interacting individuals.

A paradigmatic case of social interaction in which the performance of the action of one agent is not physically contingent upon the performance of the partner's action is communicative interaction. Consider the case of human pointing. Agent A points towards an object. Agent B turns her head to look at the object. It is only because we attribute to A the communicative intention to affect B's behaviour – for example, to inform B about the location of a certain object - that we expect A's action to be followed by B's response. The linkage between actions of the two agents is purely intentional [8]–[10] and only makes sense against the background of reciprocal communicative intention recognition [11].

In the present study we employed point-light displays to investigate whether communicative interaction influences visual discrimination of a human agent in a simultaneous masking detection task. Participants observed point-light displays of two agents (A and B) performing separate actions. In the communicative condition, the action performed by agent B (e.g., bend over to pick up something) responded to a communicative gesture performed by agent A (e.g., pointing to the object). In the individual condition, agent A's communicative action was substituted with a non-communicative, unrelated action (e.g., jumping). We hypothesized that if information picked up from communicative interaction is used to predict the partner's response, then observing A's communicative gesture should enhance visual discrimination of agent B.

## Methods

### Participants

Participants were 23 undergraduate psychology students from the University of Leuven (5 male and 18 female, mean age  = 21.1 years). They received course credits for their participation. All had normal or corrected-to-normal vision, had provided informed written consent, and were naïve with respect to the purpose of the study. The study was approved by the Ethical Committee of the Faculty of Psychology of the University of Leuven and was conducted in accordance with the ethical standards laid down in the 1964 Declaration of Helsinki.

### Stimuli

Stimuli consisted of two point-light figures, each consisting of 13 markers indicating the centre of the major joints of the actor (head, shoulders, elbows, wrists, hips, knees, and feet). Ten point-light stimuli were employed, five belonging to the *communicative condition*, five belonging to the *individual condition*. Stimuli for the *communicative condition* displayed a communicative interaction between two agents, with agent A performing a communicative gesture towards a second agent (B), who responded accordingly (e.g., A asks B to squat down, B squats down). Stimuli for the communicative condition were selected from the Communicative Interaction Database (CID, [12]), and included: ‘Get down’, ‘Pick it up’, ‘Look at that ceiling’, ‘Help yourself’, and ‘Sit down’. Stimuli for the *individual condition* were created by substituting agent A's communicative action with a non-communicative action with the same onset and duration (‘Turn’, ‘Jump’, ‘Sneeze’, ‘Lateral step’, ‘Drink’). In both the communicative and the individual conditions, the action by agent B (e.g., ‘picking something up’) was always coupled with a fixed action by agent A (‘pointing to something to be picked up’ in the communicative condition; ‘jumping’ in the individual condition).

Stimuli were constructed in accordance with the motion capture procedures described in detail by Dekeyser, Verfaillie, and Vanrie [13]. For the communicative condition, the actions of the two actors were captured at the same time, in order to guarantee that B's response matched A's communicative gesture in all respects (e.g., timing, position, and kinematics). The distance between A and B during stimulus acquisition was about two meters. A and B were always visible but the onset of A's action always preceded that of B. For the individual condition, A's action was captured while the actor was acting alone, and was then coupled with B's action, so as to maintain the same temporal structure as in the communicative interaction (i.e., A's action had the same onset and duration as in the communicative condition). Stimulus duration ranged from 3600 to 8200 ms (duration of A's actions ranged approximately from 2000 to 2600 ms; duration of B's actions ranged approximately from 2200 to 6700 ms). In both the communicative and the individual conditions, agent A and agent B remained approximately at a constant distance from the centre of the screen for the whole duration of the action and never physically touched one another. In all action stimuli (in both the individual and in the communicative conditions), they always faced each other.

#### Recognisability of the selected stimuli

In order to assess the efficacy of stimuli included in the Communicative Interaction Database, Manera and colleagues [12] examined how well each stimulus was spontaneously recognized. Communicative stimuli and non-communicative control stimuli showing two agents acting independently of each other were presented to 54 naive observers. Participants were asked, first, to decide whether the two agents (A and B) were communicating or acting independently of each other and, second, to provide a short description of the actions of both agents. Results revealed that, on average, the stimuli were correctly recognized as communicative by more than 85% of the participants; the communicative gesture of the action stimuli was correctly identified by more than 64% of the participants. For the stimuli included in the present study, the percentage of participants who correctly classified the action stimuli as communicative varied from 72% (‘Help yourself’) to 96% (‘Get down’). The percentage of participants who also correctly identified the specific communicative gesture ranged from 37% (‘Help yourself’) to 93% (‘Get down’). Control individual stimuli included in the present study were rarely classified as communicative. The percentage of participants who erroneously classified the individual stimuli as communicative varied from 23% (‘Sneeze’) to 0% (‘Jump’). The percentage of participants who correctly identified the specific individual gesture ranged from 72% (‘Sneeze’) to 98% (‘Jump’).

### Apparatus and procedure

Stimuli were displayed on a 21 inch CRT monitor (refresh rate  = 120 Hz) using MatLab (7.1 version) software. Viewing distance was 57 cm. Dots (subtending approximately 0.14 deg each) were black against a grey background and were rendered from a three-quarter view (corresponding to the 125° reference orientation used in the CID). The visual angle between the points attached to the head and the feet was about 7.15 deg. Participants were tested individually in a dimly lit and sound attenuated room.

**Figure 1:**
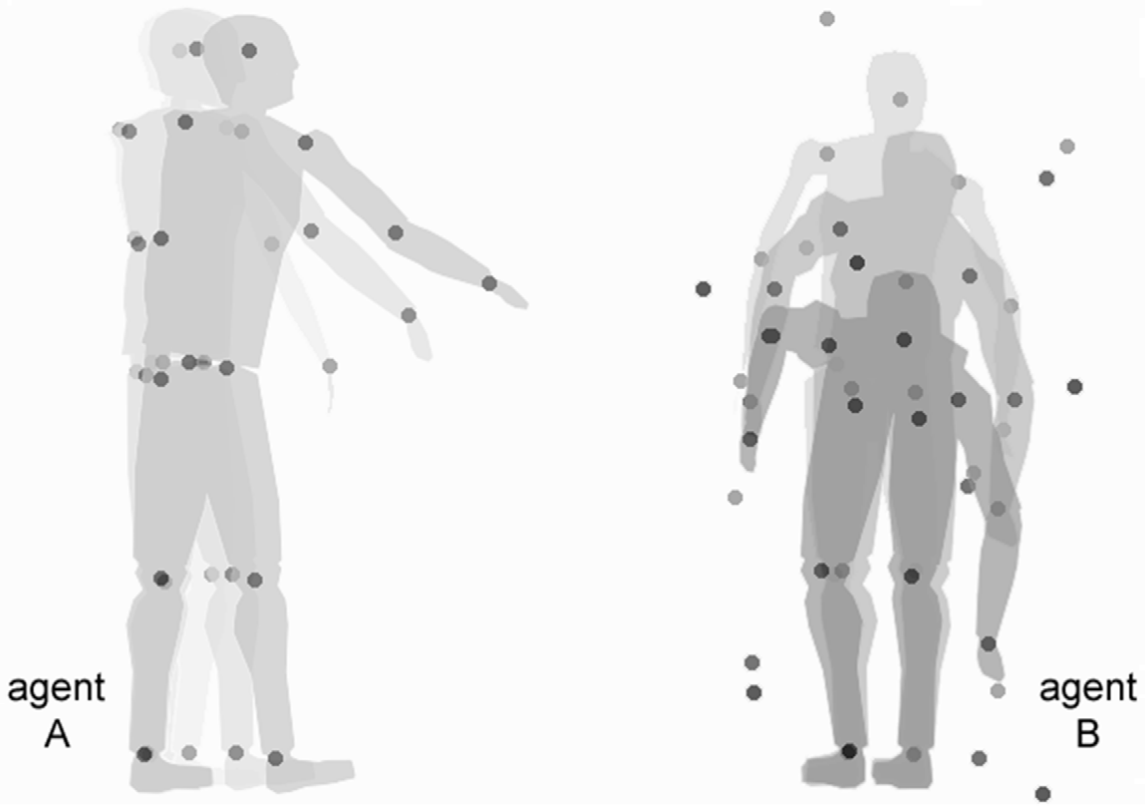
Example of a communicative signal trial. Agent A points to an object to be picked up; agent B bends down and picks it up. B was presented using limited-lifetime technique (6 signal dots) and masked with temporally scrambled noise dots. The noise level displayed is the minimum allowed in the experiment (5 noise dots). To provide a static depiction of the animated sequence, dots extracted from 3 different frames are superimposed and simultaneously represented; the silhouette depicting the human form was not visible in the stimulus display.

A two-alternative forced-choice (2AFC) paradigm was employed. Each trial consisted of two intervals, a *target* interval (containing agent B) and a *nontarget* interval (not containing agent B), with a 500 ms fixation cross (black against a grey background) in between. In the target interval, B's actions were displayed using the limited lifetime technique and masked with limited lifetime noise dots [1], [12] (see Figure 1). The limited lifetime technique was used to prevent observers from using local motion or position cues to perform the task [14]. Indeed, when dots are always visible in the same location on the actors' body, it is easier for observers to rely on local cues. The limited lifetime stimulus requires more global visual processing [1], [15]. Each signal dot was presented for a fixed duration (200 ms) at one of the 13 possible locations, then disappeared, and reappeared at another randomly chosen location. Six signal dots per frame were shown. Dot appearance and disappearance were asynchronous across frames in order to avoid motion transients from simultaneous transitions of all sampling dots. Noise dots had the same trajectories, size, and duration as the signal dots, but were temporally and spatially scrambled (they appeared in an area subtending approximately a 8.6°×14.3° region). The number of noise dots was adjusted individually for each participant during a training session (see below).

In the nontarget interval, agent B was substituted by limited lifetime scrambled dots obtained by temporally scrambling the corresponding signal action. Noise dots were also added so as to obtain the same number of dots as displayed in the signal interval. On average, positions and motions of the dots in the nontarget interval equaled those of the target interval (see also [1]). In both the target and the nontarget intervals, A was neither limited lifetime nor masked.

Observers were asked to decide which interval contained agent B as opposed to no agent. Responses were given by pressing one of two keys on a keyboard. Each participant completed four blocks of 25 trials (10 repetitions of five actions in two conditions). Each block consisted of trials of both conditions presented in a randomized order. Blocks lasted approximately seven minutes each and were separated by a rest period of two minutes. Accuracy feedback was given after each block.

#### Training session

Stimuli consisted in five actions performed by a single agent, masked with five levels of noise (5, 15, 25, 35, or 45 noise dots). The actions were different from those used in the experiment. Actions were selected from the CID and included ‘raising arms’, ‘doing aerobics’, ‘picking something up’, ‘standing up’, and ‘turning’.

Each participant completed two blocks of 25 trials (five actions by five noise levels and by two repetitions). Trials in each block were presented in a randomized order. Individual noise levels were determined by fitting a cumulative Gaussian function to the proportion of correct responses and determining the 75% threshold. The minimum noise level allowed was five noise dots (M = 22.4, SD = 17.3).

## Results

The mean proportion of correct responses was .80 (score range = .60 – .93), suggesting that the threshold estimate calculated in the training session was sufficiently accurate for most of the participants. The best detected action was “Sit down” (*M* = .85; *SD* = .14), followed by “Help yourself” (*M* = .82; *SD* = .10), “Get down” (*M* = .80; *SD* = .14) and “Look at that ceiling” (*M* = .77; *SD* = .13); “Pick it up” was the worst detected action (*M* = .74; *SD* = .12). Differences in the proportion of correct responses among the five action-stimuli reached statistical significance (within-subject ANOVA *F*_(1,22)_ = 4.09, *p* = .004). Post-hoc comparisons revealed that ‘Pick it up’ was detected significantly worse than ‘Help yourself’ (*p* = .024) and ‘Sit down’ (*p* = .028). Action detection was not related to action-stimulus duration (number of frames, r_(3)_ = .26, p = .67). In line with data concerning detection of agent's B action presented in isolation (Manera, Becchio, Del Giudice, Bara, & Verfaillie, unpublished data), these findings suggest that, independently from the context (communicative vs. individual) in which they were displayed, some actions of agent B were easier to be detected than others.

In order to compare participants' performance in the two experimental conditions, criterion (*c*) and sensitivity (*d*') parameters were extracted [16]. For each participant we calculated the proportion of *hits* (arbitrarily defined as “first interval” responses when the target was in the first interval) and *false alarms* (“first interval” responses when the target was in the second interval) in the two experimental conditions. Proportions of 0 were replaced with 0.5/N, and proportions of 1 were replaced with (N-0.5)/N (where N is the number of “first interval” and “second interval” trials).

Criterion values ranged from −.58 to .58 (*M* = .00, *SD* = .29) for the communicative condition and from −.40 to .53 (*M* = .03, *SD* = .21) for the individual condition. In neither the communicative nor the individual conditions *c* differed from zero (Single-sample T-test: communicative condition, *t* = −.02; *p* = .983; individual condition, *t* = .60; *p* = .553), thus suggesting that participants' responses were unbiased (i.e., there was no systematic tendency to respond ‘first interval’ or ‘second interval’). No difference in criterion *between* conditions was found (*F*_(1,22)_ = .20; *p* = .661).

**Figure 2:**
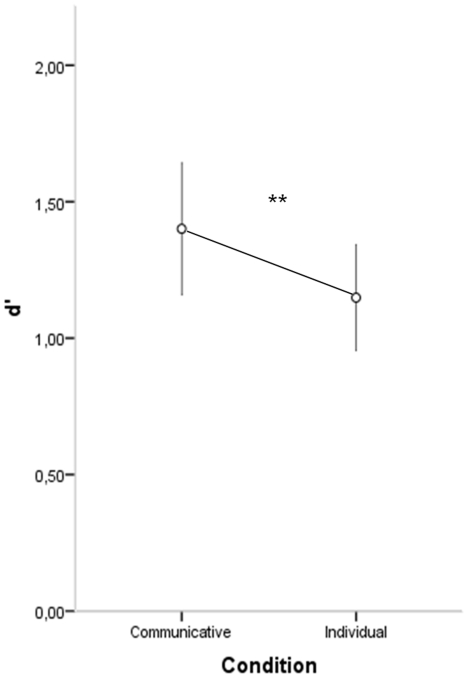
Sensitivity (d') in the two experimental conditions. Error bars represents 95% confidence intervals.

Sensitivity values ranged from .52 to 2.23 (*M* = 1.40; *SD* = .56) in the communicative condition and from .22 to 2.15 (*M* = 1.15; *SD* = .45) in the individual condition (see Figure 2). A repeated-measures ANOVA with condition (communicative vs. individual) as within-subjects factor revealed a significant effect of condition (*F_(_*_1,22)_ = 9.61; *p* = .005), with a higher sensitivity for the communicative condition compared to the individual condition.

**Figure 3:**
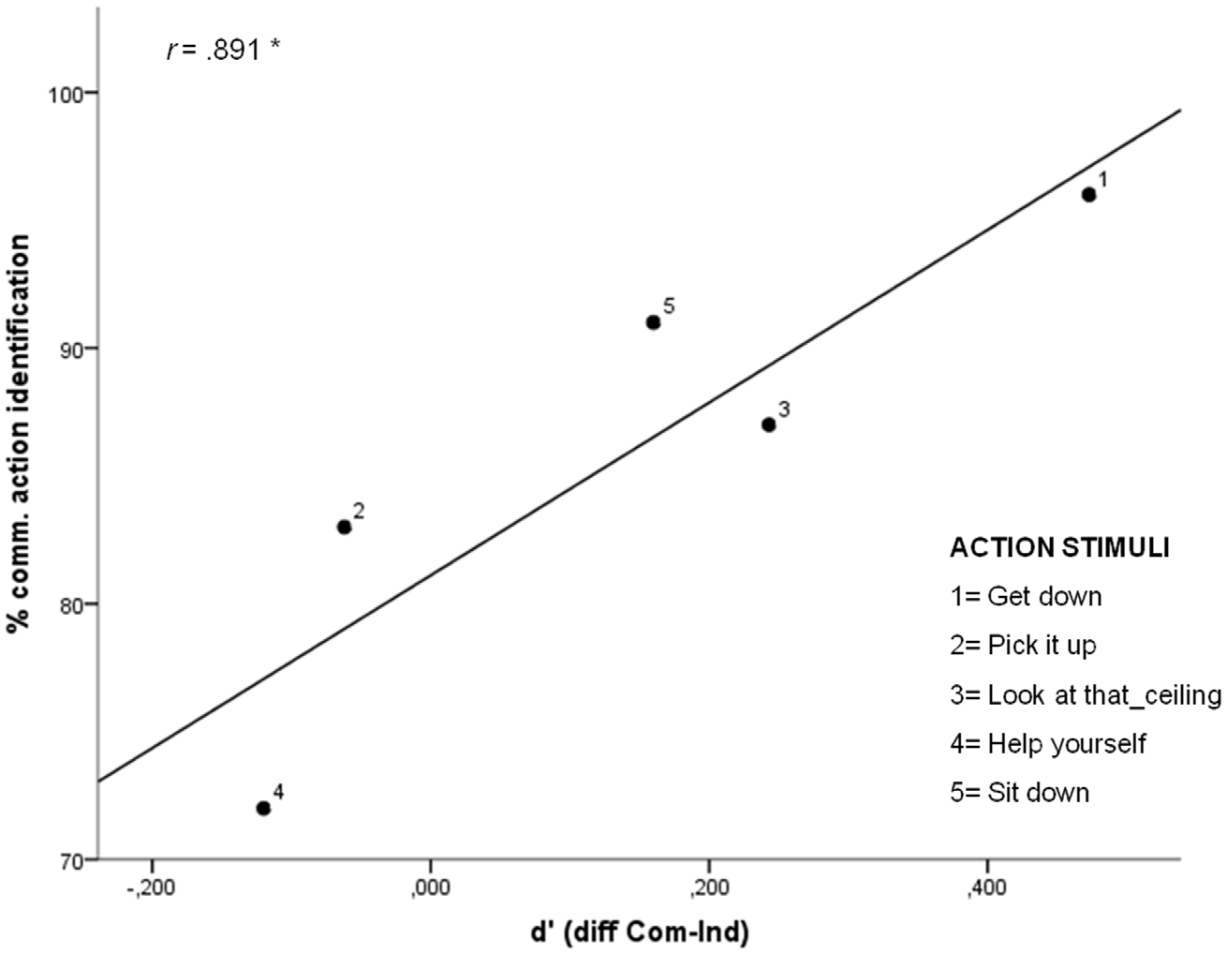
Scatter plot showing the correlation between communicative action identification and d'. Identification scores are plotted on the ordinate, and represent the percentage of participants who correctly identified A's communicative actions (normative data [12]). The difference between communicative condition and individual condition is plotted on the abscissa (*d'*). The black line represents the linear regression line fitted to the data.

To explore the role of interpersonal predictive coding further, we verified whether enhanced visual discrimination of agent B for the communicative condition was related to the recognisability of agent's A communicative gesture. If agent's A communicative gesture is used to predict B's action, then better recognition of agent's A gesture should yield better visual detection performance. To test this hypothesis, we took normative data collected to assess the recognisability of each communicative gesture (the percentage of naive participants who correctly described each gesture as communicative in Manera et al. [12]; see Methods section) and correlated this recognition index with the difference in sensitivity between the communicative and the individual condition. Recognisability of the communicative gesture performed by agent A was found to be strongly correlated with the difference between conditions (r_(3)_ = .891; *p* = .042), as shown in Figure 3.

## Discussion

Predictive coding allows humans to predict what the other person will do next [4]. In the present study we examined whether, in the context of a communicative interaction, the actions of one agent can be used to predict the actions of a second agent. We demonstrated that observing the communicative gesture performed by one agent (A) indeed enhances visual discrimination of the respondent (B) action.

How might the communicative gesture by the first agent facilitate visual discrimination of the second agent? Each action by agent B (e.g., ‘sitting down’) was always coupled with a fixed action by agent A in both the communicative (‘inviting B to sit down’) and individual (‘drinking’) condition. The associative strength between the action of agent A and the action of agent B was therefore identical in the two conditions. If facilitation simply reflected associative learning, both conditions should be equally affected by the observation of the action of the first agent and no difference should be observed. Similarly, because in both the communicative and the individual conditions the actions of the two agents were time-locked so that the onset of agent A's action always preceded the onset of agent B's action, we ruled out the possibility that our findings simply reflect onset synchronization. Clearly, observers were not only sensitive to the co-occurrence of actions, but also to the intentional link binding the actions of the two agents.

When presented with point-light displays of body movements, people can evaluate not only the kind of actions performed [17], [18] and the associated emotions [19]–[22], but also the actor's expectations [23] and intentions [24], [25]. Critically, information in point-light displays has been shown to be sufficient for clear recognition of an action as communicative, as well as for identification of the specific communicative intent in performing the action [12]. One possibility is thus that identification of agent A's communicative intent facilitated visual discrimination of a second agent by allowing observers to predict B's gesture. This interpretation is supported by the finding that visual detection performance correlated with the recognisability of A's communicative gesture: the better recognition of agent A's communicative gesture, the better visual discrimination of agent B.

An alternative explanation of the communicative versus individual effect could be that, compared to individual actions, communicative actions were more effective in triggering the participants' attention towards the action of action B. Although this consideration is plausible in general [e.g., 26,27], in our opinion it is unlikely that in the present study attentional orienting led to enhanced detection performance in the communicative condition. First, because the distance between agent A and agent B was equated in the communicative and in the individual condition, we eliminated the possibility that the communicative versus individual effect depends on the visual area participants had to keep in the focus of attention. Second, because agent A always faced agent B in both the communicative and the individual conditions, we ruled out the possibility that the reported effect is related to facing. It might be objected that despite the fact that distance and facing were the same across conditions, the gesture orientation of agent A led to enhanced discrimination performance in the communicative condition. However, if this were the case, better visual discrimination of agent B should be observed for those communicative actions that were more effective in directing attention toward a specific location in space, such us pointing gestures [28]. In contrast, no difference between the communicative and individual conditions was found for the action ‘Pick it up’ (see Figure 1 and 3), consisting of a pointing gesture toward the ground, close to agent B's foot. Furthermore, although the actions performed by agent A in ‘Help yourself’ and ‘Sit down’ are similar in driving visual attention toward the location of agent B (and of the invisible objects), enhanced visual discrimination in the communicative condition was observed for ‘Sit down’, but not for ‘Help yourself’. Because ‘Sit down’ is better recognized than ‘Help yourself’, this suggests that enhanced discrimination performance in the communicative condition was not due to attentional orienting *per se*, but to the recognition of agent A' s communicative intention.

The finding that communicative interactions improve visual detection of biological motion complements and extends previous evidence for interpersonal predictive coding [1], suggesting that the communicative gestures of one agent can serve as a predictor for the expected actions of the respondent. Future studies, using different approaches, will be necessary to understand the cognitive and neural processes underlying this phenomenon. First, functional MRI studies may help to shed light on the neural networks underpinning the facilitation effect found in the communicative condition. If visual discrimination benefits from intentional processing of A's communicative gesture – as we hypothesize here – contrasting visual discrimination in the communicative and the individual conditions should reveal differential activation in regions associated the processing of communicative intentions [29], [30]. Second, neuropsychological studies (e.g., in populations with autism spectrum disorder) might help to clarify whether processing communicative intentions is *necessary* for facilitation originating from information from the first agent. If our proposal is correct, impaired encoding of intentions should disrupt facilitation for communicative interactions, but not for physically-contingent interactions such as fighting or dancing.
